# Review of Published Laboratory-Based Aerosol Sampler Efficiency, Performance and Comparison Studies (1994–2021)

**DOI:** 10.3390/ijerph20010267

**Published:** 2022-12-24

**Authors:** James Hanlon, Karen S. Galea, Steven Verpaele

**Affiliations:** 1 Institute of Occupational Medicine (IOM), Research Avenue North, Riccarton, Edinburgh EH14 4AP, UK; 2 Nickel Institute, Rue Belliard 12, 3rd Floor, B-1040 Brussels, Belgium

**Keywords:** inhalable, respirable, thoracic, aerosol, particulates, comparison, performance, inter-sampler, EN13205, laboratory, efficiency

## Abstract

We provide a narrative review on the published peer-reviewed scientific literature reporting sampler efficiency, performance and comparison studies (where two or more samplers have been assessed) in laboratory settings published between 1994 and 2021 (27 year period). This review is a follow-up to our narrative review on the published peer-reviewed scientific literature reporting sampler comparison in workplace settings. Search terms were developed for Web of Science and PubMed bibliographic databases. The retrieved articles were then screened for relevance, with those studies meeting the inclusion criteria being taken forward to data extraction (25 studies). The most common fraction assessed has been the inhalable fraction, with the IOM sampler being the most studied inhalable sampler and the SKC Aluminium cyclone being the most studied respirable sampler from the identified relevant articles. The most common aerosol used has been aluminium oxide. It was evident that standardisation for these sampler performance experiments is lacking. It was not possible to identify any discernible trends for the performance of samplers when assessed with different aerosols. The need for more detailed and informative data sharing from authors is highlighted. This includes provision of clear identifiable information on the samplers used for testing, sampler flow rates (both manufacturer and those actually used in the study, with an explanation given of any differences), detailed information on the test aerosols used and the sampler substrate materials used. An identified gap in the literature is the potential to perform studies aimed at revaluating the performance of samplers to allow any longer-term temporal changes in performance to be assessed. One approach in advancing the field is to produce an updated protocol for the laboratory testing of samplers. This updated protocol would be beneficial for both the research and occupational hygiene community and would allow harmonised assessment and reporting of sampler comparison studies.

## 1. Introduction

Aerosol samplers are important to occupational hygienists through their role in the exposures assessment of workers to airborne contaminants. Particle size selective aerosol standards require that exposure assessment results should reflect the manner in which they are inhaled and penetrate the respiratory tract. Vincent, ISO standard 7708 and EN standard 481 [1,2,3] are describing the conventions of these size fractions, as follows:

- Inhalable fraction—this approximates to the fraction of airborne material that enters the nose and mouth during breathing, and is therefore available for deposition anywhere in the respiratory tract.
- Thoracic fraction—this is the fraction of inhaled airborne material penetrating beyond the larynx.
- Respirable fraction—this is the inhaled airborne material that penetrates to the lower gas exchange region of the lung.

As Vincent et al. reported in 1999 [1], during the preceding two decades, there had been great progress towards the setting of scientifically based criteria for aerosol measurement in the workplace and elsewhere, led by the American Conference of Governmental Industrial Hygienists (ACGIH), the International Organization for Standardization (ISO) [4] and the European Committee for Standardization (CEN) [5]. These organisations had moved towards international harmonisation of particle size-selective criteria for health-related aerosol sampling [6] and were intended to be applied in the design of new sampling instrumentation.

Liden in 2000 [7] suggested that whilst aerosol sampling had been undertaken for many years, given that there were no detailed international standards for this kind of work, it was likely that almost each laboratory would have its own specific testing protocol, which may lead to divergent results. The Aerosol Sampler Testing EXchange (ASTEX), was intended to act as a pilot project for a formal proficiency testing scheme to combat such issues. The initial aims were to provide a forum for exchanging and comparing test results, to give the members the opportunity to compare their in-house results with those obtained elsewhere and to look at ways to improve test methods and quality generally [8].

In 2002, the EN13205 standard was first published with the purpose of allowing both manufacturers and users to use a consistent approach for sampler validation and to provide a framework for assessing sampler performance in adherence to standards EN481 and EN482 [9,10]. This standard has since been updated once in 2014. The current standard consists of six separate parts, with the requirements relevant to this review being those detailed in Part 2 (performing laboratory-based tests, which is based on sampling efficiency determination) and Part 4 (which sets out the requirements for performing laboratory performance tests for concentration comparisons) [11,12].

A recent survey of aerosol sampling heads used within the metals industry revealed a wide variety of inhalable, thoracic, and respirable samplers as being commonly used [13] (Hanlon et al., 2021). This is not surprising given that a wide variety of personal samplers that have been developed [14,15]. Indeed, it was even noted in 1991 that the pressing practical need for new sampling instruments with performance characteristics matching health-related criteria had resulted in many new types of sampling devices [16]. Due to the differences in their inlet design and operational parameters (e.g., the sampling flow rate), samplers may exhibit significantly different performance characteristics [14] which can impact on data comparison, e.g., with occupational exposure limit values (OELVs) and other data sets.

In April 2019, the Nickel Institute, a global association of primary nickel producers, held a meeting with interested stakeholders regarding the development or adaptation of sampling trains to measure low levels of metals and metalloids in the workplace. Meeting participants agreed on the need for an international sampler comparison study, with the aim being to ensure exposure data, which are used in epidemiological studies, is both precise and aligned for use in the setting of OELVs. The main objective of the proposed international study is to compare currently used (and to validate any newly developed) personal samplers for measuring particulate related exposure (and more specifically metals and metalloids) in workplace settings. Sampler efficiencies for relevant aerosol size fractions of those samplers currently on the market would also be evaluated.

This literature review aims to summarise the recent published literature (1994–2021) describing sampler performance and comparison studies in laboratory settings so that current knowledge can be synthesised to inform subsequent work programmes of the international sampler comparison study. Hanlon et al. (2021) previously reported a review of the literature describing sampler comparison studies in workplace settings [13].

## 2. Methods

### 2.1. Search Strategies

The literature search was conducted in Web of Science and PubMed for studies published between 1994 and 2021 using the following search terms: (compare OR comparison* OR evaluat* OR efficiency OR performance) AND sampler* AND (aerosol OR particulate). The search year of 1994 was selected for the beginning of the search to take into account the publication of ISO 7708 (Air quality—Particle size fraction definitions for health-related sampling) in 1995.

Web of Science [17] provides access to over 21,000 journals and contains various databases which cover the journal literature of various scientific disciplines. Relevant databases included in Web of Science include MEDLINE and the Science Citation Index. Included within Web of Science are aerosol/occupational health journals such as Annals of Work Exposures and Health, Journal of Occupational and Environmental Hygiene, and the Journal of Occupational Health. PubMed [18] provides access to over 34 million citations for biomedical articles from MEDLINE and additional life science journals and is a service of the National Library of Medicine (NLM). Included within PubMed are aerosol/occupational health journals such as the Annals of Occupational Hygiene, Annals of Work Exposure and Health, and the Archives of Industrial Hygiene.

The inclusion criteria for the screening process included articles published in English; laboratory-based comparison studies; containing a comparison of at least two particulate samplers; sampler efficiency and performance tests. The exclusion criteria used for the screening process included articles that only included field (workplace) comparisons (those studies which contained both laboratory and field comparisons were included) and those that included samplers that cannot be used for personal exposure assessments of particulate aerosols in the workplace. Articles reporting vapour/gas samplers; bio aerosol samplers; real time detection instruments; articles reporting samplers used for assessing environmental air quality (particulate matter samplers) were also excluded. The inclusion/exclusion criteria were performed firstly on the title and abstract of the article. Screening of the full text of articles followed for those articles judged potentially suitable following the title and abstract screening step.

**Figure 1:**
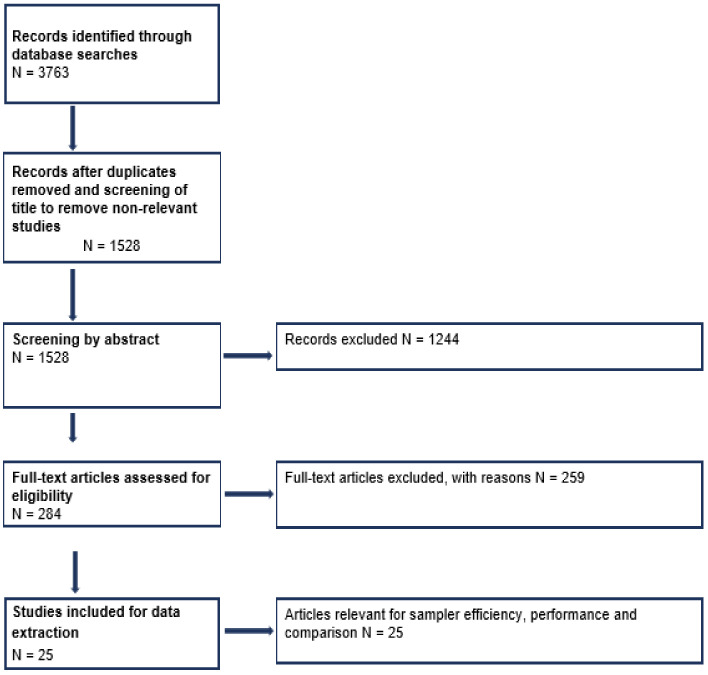
PRISMA flow diagram for laboratory-based aerosol sampler efficiency, performance and comparison studies (1994–2021).

Figure 1 summarises the review process. The initial search retrieved 3763 articles. The number of relevant articles were then reduced to 284 articles following duplicate removal and title and abstract screening. These were then subsequently screened for relevance by full text screening. Twenty-five articles were identified as relevant and were subject to data extraction.

### 2.2. Data Extraction and Collection Process

Data from the relevant articles were extracted into bespoke templates, which were developed using DistillerSR^®^ (Evidence Partners, Ottawa, ON, Canada).

For the purpose of this review, the template collected information on (where available): if the article reported a sampler performance and/or sampler comparison study, the samplers and aerosol fraction assessed, sampler mounting, sampling substrates, laboratory analysis, aerosol used, wind speeds, sampling periods, number of measurements, sampling flow rates, summary of the results and conclusions, correction factors/ratios and limitations of the publication (where stated by the authors). For the benefit of information sharing, the extracted information from DistillerSR^®^ was extracted into an Excel file and additional fields were added for completeness. These additional fields were the aim of the article, test conditions, particle aerodynamic diameter (Geometric Mean (GM) and Geometric Standard Deviation (GSD), wind direction, aerosol agglomeration, collected mass, aerosol charge, sampler specimen variability, wall deposits, temperature, pressure, correction factors for particle shape and density, determination of the test aerosol concentration, and the pumps used. This Excel file containing this information for each of the reviewed articles is available as Supplementary Materials. The experimental details that have been extracted are from some of the requirements for EN13205. Information that is presented in this Excel file are for the following headings:

- Aim of the article (this is not specified in EN13205; however, this has been added as contextual information);
- Fraction assessed;
- Samplers;
- Test conditions:
  ○ Test variables;
  ○ Particle aerodynamic diameter (geometric mean (GM) and geometric standard deviation (GSD) where reported);
  ○ Wind speed;
  ○ Wind direction;
  ○ Aerosol composition;
  ○ Aerosol agglomeration;
  ○ Collected mass and/or internally separated mass;
  ○ Aerosol charge;
  ○ Sampler specimen variability (for EN13205, this is required for the respirable and thoracic fractions);
  ○ Excursion from the nominal flow rate (for EN13205, this is required for the respirable and thoracic fractions);
  ○ Flow rates applied;
  ○ Particle collection substrates handling;
  ○ Wall deposits considered;
  ○ Weighing (balance used).
- Experimental system characteristics:
  ○ Temperature;
  ○ Pressure;
  ○ Aerosol used (mono or poly dispersed) and characteristics (GSD);
  ○ Correction factors for particle shape; SD (size distribution and concentration);
  ○ Correction factors for particle density;
  ○ Determination of test aerosol concentration;
  ○ Position and orientation of the sampler;
  ○ Pumps used and their volumetric flow deviation.

The terminology used by the current manuscript’s authors, for example, sampler name, sampling substrate (e.g., filter materials) and the purpose of the article (such as performance/efficiency/comparison), is as stated and reported by the included articles authors. The flow rates as stated by the authors are documented and where this information was not supplied this is stated. Studies have also been considered as performance studies if the original authors had compared their findings to a recognised convention.

## 3. Results

### 3.1. Samplers and Size Fractions Studied

A variety of samplers have been investigated in the identified sampler performance articles in laboratory settings (25 articles) (Table 1). The inhalable fraction has been assessed in 60% of the included articles (15 articles) in total with 11 articles assessing the inhalable fraction only. Of these articles, 12 articles included assessing the performance of the Institute of Occupational Medicine (IOM) inhalable sampler. The 37 mm Closed Face Cassette (CFC) was assessed in ten articles. The Gesamtstaubprobenahme an der Person (GSP) sampler has been assessed in six articles [19,20,21,22,23], with the Button sampler also assessed in six articles [19,20,21,24]. The seven hole sampler was assessed in two articles [22,23].

The performance of respirable fraction samplers were assessed in 56% of the identified sampler performance articles (14 articles) with 10 of these articles assessing the respirable fraction only. The most commonly assessed sampler for the respirable fraction was the SKC Aluminium cyclone (six articles; [25,26,27,28,29]). Less commonly assessed samplers for the respirable fraction included the CIP10R sampler [25,30], the Multi-Inlet cyclone [27] and a newly developed (non-commercially available) foam sampler ([26]; no further details were provided in the article).

The performance of thoracic fraction samplers has only been assessed in four of the identified sampler performance articles. One article assessed the performance of the GK2.69, the GK 4.126 (developed by MesaLabs, also known as “RASCAL”) and FSP10 samplers (article authors state that this is typically used as a respirable sampler) [31]. The other article assessed the performance of a number of thoracic fraction samplers, namely the CATHIA sampler, a modified SIMPEDS cyclone, an IOM thoracic sampler and a GK2.69 thoracic sampler [32]. The thoracic fraction has also been assessed in one article alongside the inhalable and respirable fractions [33].

**Table 1:** Samplers assessed in sampler efficiency, performance and comparison articles in laboratory settings (note—names listed as stated in publication). The flow rates are as stated in the article, in a number of articles more than one flow rate was used for a sampler and these are reported as stated.

Fraction Assessed	Samplers	Flow Rate Used in Reported Article (L/min)	Reference
Inhalable	IOM	2	[19]
	Button	4	
	GSP	2	
	37 mm CFC	2	
Inhalable	IOM	2	[20]
	Button	4	
	GSP	3.5	
Inhalable	IOM	2	[21]
	Button	4	
	GSP	3.5	
	37 mm CFC	2.25	
Inhalable	IOM	Not stated	[22]
	Seven hole	Not stated	
	GSP	Not stated	
	37 mm CFC	Not stated	
Inhalable	IOM	2	[23]
	Seven hole	2	
	GSP	3.5	
	PAS-6	2	
	PERSPEC	2	
	CIP-10l	10	
	37 mm OFC	2	
	37 mm CFC	2	
Inhalable	IOM	2	[24]
	Button	4	
	37 mm CFC	2	
Inhalable	IOM	Not stated	[34]
	37 mm CFC	Not stated	
Inhalable	25 mm CFC	2	[35]
	New inhalable (no further details)	2	
Inhalable	IOM	2	[36]
	CIP 10-l	10	
	37 mm CFC	1, 2	
	37 mm CFC with ACCU-CAP insert	1, 2	
	Button	4	
Inhalable	IOM	2	
	Button	4	[37]
	GSP	3.5	
	37 mm CFC	2	
Respirable	10 mm Dorr–Oliver cyclone	1.7	[25]
	SKC Plastic cyclone	1.9	
	Casella Plastic cyclone	1.9	
	SKC Aluminium cyclone	1.9	
	South Africa Cyclone	1.9	
	Lippmann 6 mm cyclone	2.4	
	ODPN Cyclone	0.8	
	CXF-2 virtual impactor	2.0	
	MRE 113A	10	
	CIP 10-R	10	
	C.A.Th.A sampler	2.0	
	Lippmann 12 mm cyclone	10	
Respirable	Nylon cyclone	1.5, 1.7	[26]
	SKC Aluminium cyclone	2.4, 2.7	
	New foam sampler	3.8, 10	
Respirable	Metal bodied cyclone (Casella based on Higgins–Dewell)	2.1	[38]
	Conductive plastic cyclone based on Higgins–Dewell (Casella)	2.1	
	BGI cyclone	2.1	
Respirable	10 mm nylon cyclone	1.65	[27]
	SKC Aluminium cyclone	2.67	
	Multi-Inlet cyclone	2.13	
	Big Body cyclone	2.30	
Respirable	GK2.69	4.2, 4.4	[30]
	FSP10	10, 11.2	
	CIP10R	10	
Respirable	10 mm Dorr–Oliver	1.7	[28]
	SKC Aluminium cyclone	2, 5	
	BGI4L cyclone	2.2, 4	
	GK2.69 cyclone	4.4	
Respirable	GK2.69	4.2	[39]
	FSP10	10	
	CIP10-R	9	
	GK 4.16	9	
	PPI 8	8	
Respirable	Graphite filled Casella	2.1	
	BGI stainless steel	2.1	
	BGI carbon steel	2.1	[40]
	SKC Aluminium cyclone	2.1	
Respirable	CIP10-R	10	
	GK2.69	4.2	[41]
	FSP10	10	
Respirable	10 mm Dorr–Oliver cyclone	1.5, 2, 2.5, 3	[42]
	Higgins–Dewell cyclone	1.5, 2, 2,5, 3	
Inhalable and respirable	IOM	2	[29]
	37 mm CFC	2	
	SKC Aluminium cyclone	2.5	
Inhalable and respirable	IOM	2	[43]
	IOM-MOD	2	
	Zefon coal dust cassette	2	
Thoracic	CATHIA	7	
	Modified cowled	2	[32]
	IOM thoracic	2	
	GK2.69 thoracic	1.6	
	Modified SIMPEDS	0.8	
	Modified IOM inhalable	2	
Thoracic	GK2.69	1.6	[31]
	GK4.126	2.7, 3.0, 3.3, 3.6	
	FSP10	4.0	
Inhalable, respirable and thoracic	Modified IOM sampler	2	
	37 mm CFC	Not stated	[33]
	37 mm OFC	Not stated	
	Respicon	Not stated	
	Two stage cascade impactor	10	
	IOM	2	

### 3.2. Aerosols and Conventions Used

Table 2 summaries the types of aerosols (size and how dispersed) used in the identified sampler articles, along with details of any conventions reported to have been used for assessing the sampler performance.

The most common aerosol used was aluminium oxide (7 articles; 28%), followed by coal dust (4 articles each, 16%), followed by glass microspheres and potassium sodium fluorescein which were used in three articles (12%) each.

#### 3.2.1. Agricultural Dusts

Reynolds et al. [24] compared two inhalable samplers, the IOM sampler (flow rate of 2 L/min^−1^) and the Button sampler (flow rate of 4 L min^−1^) with the 37 mm CFC (flow rate of 2 L/min^−1^) using agricultural dusts (swine, chicken and turkey) in a still air chamber (1 m^3^ Rochester style chamber with a 0.07 cm s^−1^ downward air speed) and a 1 m^2^ cross-sectional tunnel with horizontal wind speeds of 0.2–1 m/s. The SKC Aluminium cyclone (flow rate of 2.5 L/min) was also assessed. It is also worth noting for this article that the authors also performed field measurements which are discussed in our workplace sampler comparison review (Hanlon et al., 2021) [13]. Samplers were compared to each other and not to an inhalable convention and the authors concluded that the IOM sampler had the lowest coefficient of variation (i.e., the best precision) of the samplers and was least affected by wind speed changes (wind speeds of 0, 0.2 and 1.0 m/s for the sampled dusts). The authors also concluded that wind speed and the type of agricultural dust affected the obtained ratios between samplers. For example, for wind speed, the IOM/CFC Pearson correlation ratio for swine aerosols at 0 m/s was 0.67 whilst at 0.2 m/s and 1 m/s the ratios were −0.45 and 0.12, respectively. Differences between the dust types include the IOM/CFC ratio for swine at 0 m/s of 0.67, 0.81 for chicken and 0.61 for turkey. Wall deposits were not discussed as part of the author’s analysis.

#### 3.2.2. Aluminium Oxide

Seven studies used aluminium oxide as the test aerosol for sampler performance studies, with all four studies assessing the inhalable fraction.

Aizenberg et al. [19] compared the IOM, Button, GSP and 37 mm CFC samplers for the inhalable fraction using a large cross-section wind tunnel with dimensions of a height of 122 cm and a width of 183 cm (22,326 cm^2^). Aluminium oxide aerosols with medium aerodynamic diameters of 7, 29 and 70 µm were used for the testing, with wind speeds used of 0.5 and 2 m/s. The flow rates used were dependent on the wind speed used, at 0.5 m/s, the flow rates were 2 L/min for the IOM, GSP and 37 mm CFC samplers and 10 L/min for these samplers at a wind speed of 2 m/s. At both wind speeds, a flow rate of 4 L/min was used for the Button sampler with no discussion on the manufacturer recommended sampling flow rates for these samplers. At a wind speed of 0.5 m/s, when compared to the ACGIH (1999)/CEN (1993)/ISO (1995) inhalability convention, the Button sampler had a considerably better fit than the 37 mm CFC sampler. The IOM sampler was the closest to the convention, with the GSP sampler being comparable to the Button sampler. At an increased wind speed of 2 m/s, none of the samplers matched perfectly with the convention; however, the differences between samplers were not as pronounced. Of the samplers tested, the Button sampler was found to be less dependent on wind speed changes compared to the other samplers. The potential for wall deposits has not been discussed by the authors of the article.

Aizenburg et al. [21] measured the sampling efficiencies of the IOM, Button, GSP and 37 mm CFC using a simplified protocol which is based on the use of a 3D rectangular simplified torso and aluminium oxide particles (MMAD of 7, 29 and 70 µm; GSD not provided). The flow rates used were 2.5 L/min (no explanation is provided for this flow rate being selected) at a wind velocity of 0.5 m/s and 10 L/min at a wind velocity of 2.0 m/s in a large cross-section wind tunnel (height of 122 cm and a width of 183 cm). When assessed using 70 µm aluminium oxide particles, the direction-averaged sampler efficiency of the IOM sampler was 41.9% ± 5.5 at a wind velocity of 0.5 m/s and 102% ± 3 at a wind velocity of 2.0 m/s compared to the convention, which has a sampling efficiency of 50.7% for 70 µm particles. The GSP sampler under sampled compared to the convention for 70 µm particles, with sampler efficiencies of 22.9% ± 3.7 at 0.5 m/s and 29.0% ± 8.7 at 2.0 m/s. The potential for wall deposits has not been discussed in the authors of the article.

Aizenberg et al. [20] has also compared the IOM (flow rate of 2 L/min), Button (flow rate of 4 L/min) and GSP (flow rate of 3.5 L/min) samplers in their later publication for sampler efficiencies when measuring large particles using a closed-loop, open cross-section wind tunnel. The flow rates used were the manufacturers nominal flow rates as stated by the authors (2 L/min for the IOM sampler, 4 L/min for the Button and 3.5 L/min for the GSP sampler). At a wind velocity of 1 m/s and a particle size of 70 µm, the IOM sampler matched the inhalability (ACGIH, 2000) convention well, whilst at a wind velocity of 0.5 m/s, the direction averaged sampler efficiency increases when the particles sizes are near the upper boundary of the inhalable fraction and beyond. For the Button and GSP sampler efficiencies, these did not essentially change when the particle size was decreased and the wind velocity did not significantly affect the sampling efficiencies (*p* = 0.23 for the Button sampler and *p* = 0.43 for the GSP sampler). It is worth noting that the aim of this article was to assess a simplified test protocol developed by the authors in their earlier publications (i.e., [21]) and the authors concluded that their new wind tunnel design could enhance the inhalable convention to be used beyond 100 µm. The potential for wall deposits has not been discussed by the authors of this article.

The sampling efficiencies of the IOM sampler, the seven-hole sampler, the GSP sampler and the 37 mm CFC have been evaluated in low wind conditions of 0.5 m/s [22]. The aluminium oxide used was from a narrow-fraction aluminium oxide grinding powder, Duralum (no discussion on the specific diameter size used in the article). The flow rates of the samplers are not stated. Aerosol chambers of approximately 1 m^2^ and an overall height of approximately 2 m were used, with a wind speed of 0.5 m/s. At low wind speeds, the IOM sampler agreed well with the inhalability convention (ACGIH, 1985/CEN, 1993 [9]/ISO, 1995 [4]). The potential for wall deposits has not been discussed by the authors of this article.

A number of inhalable samplers (IOM at a flow rate of 2 L/min, seven-hole at a flow rate of 2 L/min, GSP at a flow rate of 3.5 L/min, PAS-6 at a flow rate of 2 L/min, PERSPEC at a flow rate of 2 L/min, CIP-10l at a flow rate of 10 L/min, 37 mm OFC at a flow rate of 2 L/min and 37 mm CFC at a flow rate of 2 L/min) were used by Kenny et al. [23] for assessing the sampler performance compared to a draft CEN (1995) standard for developing workplace samplers. Graded narrow-fraction aluminium oxide was used with near monodisperse test aerosols produced with aerodynamic diameters of 1 to 25 µm. In terms of wall loses, the authors discuss that only particles that landed on the filter/foam substances are intentionally included, except for the IOM sampler although there is no further elaboration of this point by the authors. Only a summary of the results are presented in the publication as the authors discuss that a detailed publication was in preparation (this has not been identified from the PubMed and Web of Science searches). A large-section wind tunnel and wind speeds of 0.5, 1 and 4 m/s were used. For sampler precision at 0.5 m/s and 1.0 m/s, the GSP sampler was identified by the authors as being the most precise, with the seven-hole sampler being the least precise compared to the inhalable convention. At 4.0 m/s, the PERSPEC sampler was the most precise with the 37 mm OFC being the least precise. At higher wind speeds (4 m/s), none of the samplers performed well.

Sleeth and Vincent [37] investigated the sampler efficiencies of four inhalable samplers with aluminium oxide as the test aerosol. These were the IOM sampler at a flow rate of 2 L/min, the Button sampler at a flow rate of 4 L/min, the GSP sampler at a flow rate of 3.5 L/min and the 37 mm CFC sampler at a flow rate of 2 L/min. At a wind speed of 0.2 m/s and above, the samplers matched the inhalability convention relatively well. However, below this wind speed at a wind speeds of 0.10, the samplers had a greater sampling efficiency than the inhalability convention. The authors concluded that there was an increase in the sampling efficiency with lower wind speeds.

Lee et al. [41] investigated the sampling performance of the CIP10R, GK2.69 and FSP10 samplers for the respirable fraction using aluminium oxide along with ammonium fluorescence. For aluminium oxide, the CIP-10R authors compared these samplers with a 10 mm nylon cyclone and HD cyclone. From the net mass ratio results, the CIP10-R sampler collected 5–9 times that for the nylon cyclone and 3–6 times that of the HD cyclone; the GK2.69 sampler collected three and two times more dust, respectively, than the 10 mm cyclone and the HD cyclone, and the FSP10 sampler collected 7–11 times that of the nylon cyclone and 5–7 times that of the HD cyclone.

#### 3.2.3. Ammonium Fluorescein

Lee et al. [31] assessed the performance of the GK2.69 cyclone (flow rate of 1.6 L/min), the GK4.126 cyclone (flow rate of 3.5 L/min) and the FSP10 cyclone (flow rate of 4.0 L/min) using ammonium fluorescein particles (monodisperse 2.1–14.6 µm aerodynamic particle size GSD 0.1–0.5) for the thoracic fraction. The authors first of all determined the flow rates needed for obtaining a sampling efficiency of 50% for a particle size of approximately 10 µm. Experiments were performed at two laboratories, NIOSH and HSL, with NIOSH using monodisperse ammonium fluorescein and HSE using ballotini glass beads (no explanation is provided for the differences in aerosols used). No description of the chambers and wind speeds are provided. The obtained d50 (50% penetration cut-off value) values using ammonium fluorescein particles were 9.7 µm for the GSK2.69, 9.8 µm for the GK4.126 and 10.9 µm for the FSP10 with the flow rates used. Wall deposit issues were not reported in the article. Along with tests using ballotini glass beads (discussed in Section 3.2.4), the authors therefore concluded that the GK4.126 performance demonstrated minimum bias to the ACGIH (2014)/CEN (1993)/ISO (1995) [2,9,44] thoracic convention at a 3.5 L/min flow rate.

Lee et al. [41] investigated that the performance of three respirable samplers compared to the ISO/CEN/ACGIH respirable convention (years not supplied). The samplers assessed were the CIP 10-R sampler (flow rate of 10 L/min), the GK 2.69 sampler (flow rate of 4.2 L/min) and the FSP10 sampler (flow rate of 10 L/min). These samplers when faced with ammonium fluorescein monodisperse particles, overestimated exposure compared to the convention.

#### 3.2.4. Arizona Road Dust

The GK2.69, FSP10 and CIP10R samplers for the respirable fraction were assessed for collection efficiencies for Arizona Road Dust (Stacey et al. [30]). The flow rates used for the article were as follows: GK 2.69 at 4.2 L/min (manufacturer flow rate) and 4.4 L/min (NIOSH proposed flow rate), FSP 10 at 10 L/min (manufacturers flow rate) and 11.2 L/min (NIOSH revised flow rate), and 10 L/min (manufacturer flow rate) for the CIP10R. The SIMPEDS sampler was also used as a reference. The authors used an aerosol chamber and tested the samplers in calm air conditions with a wind speed of ~0.4 cm/s. Two grades of ISO (12103-1, 1997) Arizona Coal Dust were used, these being an ‘ultra-fine’ grade (0–10 µm particle size) and a ‘medium’ grade (0–80 µm particle size). For the collection of respirable crystalline silica from Arizona Coal Dust, the CIP10R and the SIMPEDS samplers collected the lowest proportions of quartz, with the GK2.69 sampling the highest. For differences between samplers for the collection of ultrafine (0–10 µm particle size) and medium (particle size 0–80 µm) Arizona Road Dust were not significantly different due to the variability of the samplers (*p* > 0.01). Alongside other sampling performed, adjusting the flowrate of the FSP10 sampler to 11.2 L/min improved the sampler performance, whereas the difference between the two flow rates (4.2 and 4.4 L/min) for the GSK2.69 sampler was considered unlikely to have an impact on the sampler performance. The effect of wall deposits has not been considered by the article authors.

The GK2.69 (low rate of 4.2 L/min), CIP10-R and FSP10 (flow rate of 10 L/min) alongside the GK4.16 (RASCAL) (flow rate of 9 L/min and PPI 8 (flow rate of 8 L/min) respirable samplers have been assessed for the performance when sampling dust of varying particle size, including Arizona Road Dust (coarse grade, ISO 1997, 0–180 µm diameter) [39]. A dust tunnel (12 m long, height of 1 m and a 1.5 m width cross section) and wind speeds of 1 and 2 m/s were used for the sampler comparison. The GSD of the Arizona Road Dust used was 2.65 g cm^−3^, with a GM of 22 µm at 1 m/s and 17.4 µm at 2 m/s. For the collection of different grades of the road dust in calm air conditions, the authors concluded that the relative collection of the samplers is consistent when the aerosols had a smaller median aerodynamic particle size (<4 µm). Other dusts were also used in this article, namely coal and quartz (these results are discussed in Section 3.2.5 and Section 3.2.11).

#### 3.2.5. Coal Dust

Görner et al. [25] compared fifteen respirable samples using an aerosol of ground coal dust. Only those samplers with flow rates of ≤10 L/min are considered here; a number of samplers compared in this article were used at flow rates above 10 L/min and thus would not be used as personal samplers. The samplers considered here are as follows: 10 mm Dorr–Oliver (1.7 L/min and 1.5 L/min), SKC cyclone (1.9 L/min), Casella cyclone (1.9 L/min), SKC Aluminium cyclone (1.9 L/min and 2.2 L/min), the South Africa cyclone (1.9 L/min and 2.5 L/min), the Lippmann 6 mm cyclone (2.4 L/min), ODPN cyclone (0.8 L/min), the CXF-2 virtual impactor (flow rate of 2.0 L/min), the MRE 113A (2.5 L/min), the CIP 10 (10 L/min) and the Lippmann 12 mm cyclone (10 L/min). The potential for wall deposits has not been discussed by the authors of this article.

The authors conclude that for the samplers, there is not an “ideal” sampler that can be used as a reference sampler for every situation for the aerosol tested. In terms of sampler performance, the majority of the samplers met the accuracy performance criteria of 80% or above for the standard (CEN (1993)-ISO (1995)-ACGIH (1994–1995) [4,9]. However, some samplers did require flow rate modifications to meet this criteria The Dorr-Oliver nylon cyclone had a modified flow rate of 1.5 L/min (nominal flow rate of 1.7 L/min), the SKC Aluminium cyclone had a modified flow rate of 2.2 L/min (nominal flow rate of 1.9 L/min) and the South Africa cyclone had a modified flow rate of 2.5 L/min (nominal flow rate of 1.9 L/min) [25]. The authors also recommend that sampler bias maps should be used for determining over/under sampling of aerosols.

Soo et al. [28] compared the 10 mm Dorr–Oliver cyclone (flow rate of 1.7 L/min), the SKC Aluminium cyclone (flow rate of 2.5 L/min), the BGI4L cyclone (flow rate of 2.2 and 4 L/min) and the GK2.69 cyclone (flow rate of 4.4 L/min^)^ for assessing quartz collection in a calm air chamber (no further details on the chamber and wind speed are provided) using quartz from Pittsburgh seam coal dust. The BGI4L cyclone had a higher internal surface deposit mass fraction, but this was not significant compared to the other samples for the polystyrene cassette. The GK2.69 cyclone, however, significantly had a lower interior surface deposited quartz mass fractions (*p* < 0.05). The authors conclude that interior surface deposits in polystyrene cassettes needs to be considered in the analysis.

The IOM inhalable sampler, a modified IOM sampler with an interchangeable isokinetic nozzle (IOM-MOD), a Zefon Coal Dust Sampling Cassette (directly in line and facing the wind tunnel) and a Zefon Coal Dust Sampling Cassette at 90° to the flow and facing the tunnel floor, have been compared for sampling coarse coal dust [43]. The authors used a long wall gallery (38 m length, 6 m width and height of 3 m) with two types of coal dust used (Keystone Mineral Black (Fine) 325BA and custom-ground coal dust with a 53 µm volume mean diameter). A flow rate of 2 L/min^−1^ was used for the samplers. A significant difference was observed between the IOM-MOD and Zefon coal cassette mass concentrations when a high air velocity (2.85 m/s) was used with fine dust (<10 µm, 63% by volume) and coarse dust (<10 µm, 14% by volume) and in low air velocity of 0.5 m/s with coarse dust. Comparing the sampler repeatability only showed that the IOM-MOD sampler demonstrated a coefficient of variation of less than 10%. The authors conclude that for the assessed samplers, the IOM-MOD sampler may improve the dust assessment over “off-the-shelf” samplers.

The GK2.69 (low rate of 4.2 L/min) and FSP10 (flow rate of 10 L/min) alongside the GK4.612 (flow rate of 9 L/min), CIP10R (flow rate of 10 L/min) and PPI 8 (flow rate of 8 L/min) respirable samplers were compared using Pittsburgh Coal Dust [39]. This publication also used Arizona Coal Dust (Section 3.2.4). Ten tests were performed using Pittsburgh Coal Dust at 1 m/s^−1^, with three facing, four side-on and three rotating tests. One set of tests were performed at a wind speed of 2 m/s, results are presented in Table 3 when the samplers were compared to a reference SIMPEDS sampler. Wall deposits have not been considered as part of the analysis. Particle losses due to the internal wall of the sampler are discussed for the GSP sampler as part of the analysis.

#### 3.2.6. Glass Microspheres

Four studies used glass spheres as the test aerosol for sampler performance. Aerosol penetration has been measured by Maynard [32] for six thoracic samplers using a polydisperse aerosol of glass microspheres. These were the CATHIA sampler (flow rate of 7 L/min), a modified cowled sampler (with a foam plug insert) at a flow rate of 2 L/min, the IOM thoracic sampler (modified IOM inhalable sampler with foam plug insert) with a flow rate of 2 L/min and the GK2.69 thoracic cyclone with a flow rate of 1.6 L/min. A calm air chamber and a low velocity wind tunnel and wind speeds between 0.5 and 4 m/s were used in the article. When compared to the thoracic convention (CEN (1993)/ISO (1995) [2,9]), the modified SIMPEDS cyclone and the GK2.69 performed well. The IOM modified sampler when operated at wind speeds above 0.5 m/s would also be close to the thoracic convention. Potential bias was identified by the authors for the CATHIA sampler as this oversampled with respect to the convention. The potential for wall deposits has not been discussed in the article.

In addition to experiments using ammonium fluorescein particles, Lee et al. [31] performed experiments using polydisperse glass sphere particles (Ballotini glass beads) for the GK4.126 cyclone for the thoracic fraction (Lee et al. discuss that the GK4.126 cyclone with a flow rate of 8.5 L/min was developed for the respirable fraction). Flow rates of 2.7, 3.0, 3.3 and 3.6 L min^−1^ were investigated. No large difference in bias (>10%) was found for particle size distributions that had a MMAD < 20 µm and a GSD > 2.0, irrespective of the flow rate. Along with the ammonium fluorescein experiments, the GSK4.162 demonstrated minimum bias to the ACGIH (2014)/CEN (1993)/ISO (1995) [2,9,44] thoracic convention at a 3.5 L/min flow rate. In this article, wall deposits have not been discussed and no information is included on the wind speeds used.

Maynard et al. [38] assessed three respirable samplers, these being the BGI cyclone sampler (plastic and metal, no more information is supplied for the sampler type), a metal bodied cyclone and a conductive plastic cyclone (both Casella cyclones) using glass microspheres (2.5–20 µm nominal mass distribution). The flow rates were 2.1 L/min instead of 1.9 L/min, as a flow rate of 2.1 L/min was previously found to lead to a better agreement with ISO/CEN/ACGIH (CEN 1993) sampling convention. The sampling characteristics of the BGI and Casella plastic cyclones were similar whereas the Casella metal cyclone was significantly different. The metal Casella cyclone had a significantly higher d50 (5.05 µm), whilst the Casella plastic and BGI cyclones were lower (pooled d50 average of 4.59 µm). Wall deposits were not considered in the article. However, an issue was noticed regarding the tolerance error of the input slot width of the Casella cyclone, which has had an effect on the sampling efficiencies of the Casella metal cyclone. The acceptable tolerance limit is ±3%, however this was found to be 13% due to a machining error. The authors of the article reported that this has been subsequently rectified.

Görner et al. [36] measured the sampling performance of six inhalable samplers. These were the IOM sampler (flow rate of 2 L/min), the CIP 10-l v1 and v2 samplers (flow rate of 10 L/min), the 37 mm CFC (flow rate of 1 and 2 L/min), the ACCU-CAP sampler (flow rate of 1 and 2 L/min) and the Button sampler (flow rate of 4 L/min). A vertical calm air chamber and a horizontal wind tunnel at a wind speed of 1 m/s were used. When compared to the inhalable convention (CEN (1993)-ISO (1995)-ACGIH (1994–1995), the IOM and CIP 10-l v2 had a high sampling efficiency to the convention. The CIP 10-l v1 and Button samplers had a lower sampling efficiency when compared to the convention, whereas the 37 mm CFC sampler had the poorest efficiency compared to the convention. However, differences were observed between calm air and moving air with the sampling efficiency curves different for all of the samplers. The potential for wall deposits has been discussed in the article, with the sampling efficiency of the 37 mm CFC being improved by also an ACCU-CAP insert. This helps to prevent inner wall loses inside the 37 mm CFC.

#### 3.2.7. ISO Test Dust

ISO test dust with a reported density of 1.56 g/cc (fine, medium and coarse) has been used by O’Shaughnessy et al. [29] for comparing the IOM sampler (2 L/min flow rate), the 37 mm CFC (2 L/min flow rate and the SKC Aluminium cyclone (2.5 L/min flow rate) in a still air chamber (downward velocity of 0.07 cm/s for air) and a wind tunnel (wind speeds of 0.2 m/s and 1.0 m/s) for producing bias ratios between the samplers. Using the ISO test dusts, the average cassette/IOM sampler ratio was not significantly different than 1 at wind speeds of 0.2 and 1.0 m/s. The bias ratios for different wind conditions (still air chamber, 0.2 and 1.0 m/s) were not significantly different for the 37 mm CFC/IOM sampler (*p* = 0.127) and the cyclone/IOM bias ratio (*p* = 0.127) for the ISO test dust.

#### 3.2.8. Organic Dust

Organic dust collected from a grain elevator has been used in a laboratory setting by the authors for comparing the IOM sampler (2 L/min flow rate), the 37 mm CFC (2 L/min flow rate and the SKC Aluminium cyclone (2.5 L/min flow rate) in a still air chamber (1 m^3^ Rochester-style chamber) and wind tunnel with a 1 m^2^ cross-sectional area (at 0.2 and 1.0 m/s) and producing bias ratios between the samplers [29]. This dust was used by the authors alongside the ISO test dust to show differences between different dusts. A significantly lower bias was obtained using the organic dust when compared to the ISO test dust for the cyclone/IOM bias ratio (*p* = 0.001) and the CFC/IOM bias ratio (*p* = 0.033).

#### 3.2.9. Polystyrene

Higgins–Dewell-type respirable cyclones (brass Casella, graphite filled polypropylene Casella, BGI stainless steel, BGI carbon steel and SKC Aluminium cyclone) were compared using a modified TSI APS method which is used to measure the particle size distribution [40]. Polydisperse aerosols used were generated from different solutions, which had polystyrene concentrations between 0.1–5.5%. A chamber of 1 × 1 × 1 m^3^ was used with an estimated air velocity of below 0.1 m/s. A flow rate of 2.1 L/min was used for all the cyclones. The authors conclude that for the accurate determination of cyclone penetration, particle loading also needs to be considered and each sampler has a unique cutoff diameter and slope for the penetration curve. It is also concluded that modifications performed by manufacturers can also have an effect on cyclone penetration.

#### 3.2.10. Potassium Sodium Tartrate

Three studies have been identified which have used potassium sodium tartrate (PST) as the test aerosol to assess the performance of samplers.

The laboratory performance of the nylon cyclone (flow rate of 1.7 L/min and 1.5 L/min), SKC Aluminium cyclone (flow rate of 2.5 L/min and 2.7 L/min) and a a newly developed foam sampler (flow rates of 3.8 and 10 L/min) has been assessed by Chen et al. [26]. The potassium sodium tartrate used had count median diameters of either 3 or 8 µm (GSD of 1.6). Micrometer-sized liquid dioctylphthlate was also used by the authors. The dimensions of the testing chamber and wind speeds used are not discussed. For meeting the ISO (1993)/CEN (1992)/ACGIH (1998) [45,46] respirable convention 50% cut-off size, sampling flow rates of 1.5 L/min and 2.7 L/min were the optimal rates for the nylon cyclone and the SKC Aluminium cyclone, respectively. The new foam sampler was also found to meet this requirement. The potential for wall deposits has not been discussed in the authors of the article.

Bartley et al. [42] evaluated the performance of the 10 mm nylon Dorr–Oliver cyclone and the Higgins–Dewell cyclone with PST particles (mass median diameter of 4 µm with a GSD of 2.2). The initial flow rates or nominal flow rates of the samplers is not discussed. A low-speed wind tunnel with a mean air speed of 0.54 m/s was employed. The authors found that the bias between the two samplers were nearly identical (<10% over a wide size distribution range). The sampling efficiency dropped towards zero more sharply than the convention (ACGIH/ISO/CEN). For matching the convention, the recommended flow rates provided are 1.7 L/min for the Dorr–Oliver cyclone and 2.2 L/min for the Higgins–Dewell cyclone. Wall deposits have not been discussed.

The performance of four respirable samplers, the 10 mm nylon cyclone (1.65 L/min flow rate), the SKC Aluminium cyclone (2.67 L/min flow rate), the Multi-Inlet (MI) cyclone (2.13 L/min flow rate) and the Big Body (BB) cyclone (2.30 L/min flow rate) has been assessed using micrometre-sized PST particles (Count Median Diameter (CMD) of 3.5 and 7.45 µm) [27]. The authors explain that these flow rates have been used to give a 4 µm cut size. The authors also used dioctyl phosphate as a liquid challenge aerosol and sodium chloride as a solid challenge aerosol; these results are not discussed in detail in the article. These flow rates, which are different from those of the manufacturers (not supplied), were used by the authors as these gave a 4 µm cut-point. The wind speed(s) used is not discussed in the article. Wall deposits are considered by the authors and the authors explain that PST has been used to investigate if the aerosol size affects side wall deposition and that underestimation may occur due to this wall deposition. For small particles, the Nylon and SKC cyclone separation efficiency curves were sharper than the respirable convention previously reported. MI and BB separation efficiency curves had a better fit to the ACGIH/CEN/ISO respirable convention. For large particles, the MI and BB cyclones matched the convention. For the SKC cyclone, there was a notable increase in aerosol penetration between 4 and 7 um.

#### 3.2.11. Quartz

Alongside field tests (not discussed here), Teikari et al. [33] investigated the performance of inhalable, respirable and thoracic samplers in a 143 m^2^ outdoor environmental chamber with an airflow of around 0.1 m/s. The samplers included in the assessment were the IOM sampler, a modified IOM sampler (with porous plastic foam insert for sampling respirable and thoracic fractions), the 37 mm CFC and OFC, the Respicon and the two-stage cascade impactor. The recommended sampling flow rates from the manufacturers were used (no further information provided). For the inhalable fraction in the laboratory tests, the IOM sampler and the modified IOM sampler measured similar concentrations. However, both the Respicon and 37 mm CFC measured around 50% of that of the IOM samplers for the inhalable fraction. The two-stage cascade impactor and the Respicon measured oversampled compared to the modified IOM sampler for the respirable fraction. The modified IOM sampler also sampled the highest concentrations for the thoracic fraction with the RespiCon sampling the lowest concentrations. Wall deposits have not been discussed as part of the author’s analysis.

#### 3.2.12. Refractory Mineral Dust

One article was identified that used refractory mineral dust (mainly Mullite with a mean particle size of 14.9 µm with 10% crystalline silica) for the GK 2.69, FSP10 samplers, alongside the GK 4.162 (RASCAL) and PPI 8 respirable samplers [39]. This publication also involved Arizona Road Dust in which more details on the set-up and sampler flow rates are discussed (see Section 3.2.4). The results are presented for trend lines using X-ray diffraction. When compared with a SIMPEDS reference sample, the slopes were 1.05 for the GK 2.69 sampler, 1.04 for the GK 4.16 sampler, 1.04 for the PPI8 sampler and 0.97 for the FSP10 sampler.

#### 3.2.13. Sodium Fluorescein

Two articles were identified that used sodium fluorescein as the test aerosol. The IOM sampler, Seven-hole sampler, the GSP sampler, the PAS-6 sampler, the PERSPEC sampler, the CIP10-l sampler and the 37 mm OFC [23] were evaluated with the inhalable convention using monodisperse sodium fluorescein (alongside aluminium oxide). The results for using monodisperse sodium fluorescein for small particles were found to be of fair agreement compared to that for aluminium oxide (discussed in Section 3.2.2). The authors discuss that sodium fluorescein was also used as the smallest grade for the aluminium oxide used (Aloxite) may have contained agglomerates and affected the sampler efficiency. Further information on this article has been previously discussed in Section 3.2.2.

Kalatoor et al. [35] used sodium fluorescein (physical diameters of 13.5, 20 and 30 µm) for assessing a new inhalable sampler (name not supplied; 2 L/min flow rate) and compared the sampler efficiency with the 25 mm CFC cassette. This was performed using flow pattern visualisation and a horizontal high velocity aerosol wind tunnel with an air velocity of 1–3 cm/s used. The filter deposition uniformity of the new sampler was more than twice that of the 25 mm CFC at isoaxial orientation (θ = 0°, Relative Standard Deviation: 19.2% versus 44.6%). At 45° there was a preference for the new sampler (Relative Standard Deviation: 19.7% versus 28.2%), whereas at 90° orientation there was no noticeable differences between the two samplers. The loss of particles from wall deposits is discussed but is not included in the analysis.

#### 3.2.14. Test Aerosol (Not Stated)

Smith and Bartley [34] used a ‘test aerosol’ of 7 µm (MMAD) to evaluate the sampler efficiency of the IOM (conductive material) and 37 mm CFC samplers (conductive/non-conductive) using conductive and non-conductive manikins and samplers. A wind speed of 0.5 m/s was used in a 183 cm wide by 122 cm high cross section wind tunnel. The authors concluded that sampler and manikin conductivity had an effect of sampler efficiency, with the highest efficiency observed for the conductive samplers (no direct comparison between the samplers is discussed). Using a conductive manikin also resulted in a higher sampler efficiency than a non-conductive manikin. The effect of wall deposits is not discussed.

### 3.3. Correction Factors

Only two of the identified articles provided correction factors. One article has provided correction factors from using aluminium oxide and sodium fluorescein test aerosols [23]. These correction factors were provided for obtaining a satisfactory sampler performance for reducing the sampler bias at wind speed of 0.5 and 1.0 m/s. The correction factors at 0.5 m/s are 1.15 for the CIP10-l and 37 mm OFC samplers, 1.0 for the PAS-6, seven-hole, 37 mm CFC, GSP, PERSPEC and IOM (filter only) samplers. For the IOM (filter and cassette) a correction factor of 0.9 is supplied. The correction factors at 1.0 m/s are 1.15 for the CIP10-l and 37 mm OFC samplers, 1.25 for the PAS-6, 1.2 for the seven-hole and 37 mm CFC, 1.0 for the GSP, PERSPEC and IOM (filter and cassette). No correction factor is supplied for the PERSPEC sampler as the inlet losses were not recovered.

The second article supplied correction factors for using a polydisperse polystyrene aerosol [40]. Correction factors in the range of 0.95–1.1.5, dependent on the particle size for three respirable cyclones, namely the Casella, BGI and SKC cyclones were used for obtaining unity penetration between 1.1–1.7 µm. The specific correction factor for each sampler is not discussed.

### 3.4. Other Aspects Identified for Consideration

Within the Excel file (available as Supplementary Materials) which contains the extracted information from the articles, there are number of aspects which are not discussed in the proceeding sections. These aspects are further discussed in the discussion section (Section 4).

Information on the experimental set up used for testing could be limited; for example, details on the chambers used (such as dimensions) has not been described in articles (i.e., [28,32]). For test variables, information on particle aerodynamic diameter was lacking in some articles, wind direction was not discussed in a number of the articles, information on aerosol agglomeration and the collected mass and/or internally separated mass was lacking in most of the articles. The inclusion of wall deposits was also not considered in most of the articles.

For experimental system characteristics, information was lacking in most of the articles for the temperature and pressure. Information was also lacking on any correction factors used for particle shape and/or density. Half of the articles also included no discussion on the determination of the test aerosol concentration, and information on the pumps used was lacking in the majority of the articles (16 articles).

## 4. Discussion

### 4.1. Summary of the Studies

This present article provides a review of laboratory based sampler performance studies (where more than one sampler has been assessed) in the peer-reviewed literature between 1994 and 2021 (a 27-year period).

Information on the most common particulate fractions and the samplers used for the sampler performance studies is provided in Table 1 and Table 2. From this review, it is evident that with the nature of the testing performed in the studies, the differing samplers being assessed and the different conventions being used, it is difficult to compare the performance of samplers across the various studies.

Themes can however be extracted from the reviewed articles which are outlined as follows and it is essential that all aerosol sampler stakeholders (manufacturers, occupational hygienists, industry and academia) reflect upon these to further advance the field.

### 4.2. Terminology Used

In this review, we have reported the results as described by the article authors. This includes, for example, the sampler name and sampling substrates, although in some cases limited or no information is supplied on the sampling substrates used.

For sampler name, it needs to be clearer which sampler is being tested, for example, in the case of samplers under development these should be named so to allow data tracking of the sampler. This is the case for one sampler that was developed by Chen et al. [26]. It is also recommended that product codes for the samplers should at least be supplied by authors for traceability and for future reference. For sampler performance studies, it also needs to clear if the authors are looking at performance or efficiency and for an explanation to be provided. Where possible, these definitions provided in EN13205 and EN1540 should be used by authors. For example, the definition used for sampler efficiency in EN1540 is “*<aerosol sampler> for each aerodynamic diameter of a particle, relative fraction of the concentration of airborne particles transported from the undisturbed air to the collection substrate for analysis*”. In some cases for performance and efficiency, it can be unclear what is meant by the authors, this should be stated in the article along with the aim of the performance or efficiency article.

### 4.3. Conventions Used

Of the twenty-two articles, ten articles do not mention use of conventions in their assessment. For those twelve articles that discussed conventions, due to the differing years that the articles have been published, the conventions used also varies. Typically, the conventions used are those published by ISO, CEN and ACGIH with varying years used.

There are cases where articles provide references to conventions however, the years are unclear. Bartley et al. [42] discuss the convention; however, it is unclear which one has been used for the analysis. Chen and Huang do not supply any information in the reference list [27].

When conventions are used for sampler performance, full citation details should be provided by the authors.

### 4.4. Discussion on Experimental Set-Ups

The Excel file provided as Supplementary Materials contains data extracted on the test conditions, the test variables and experimental system characteristics for each article. It is worth noting that for the various parameters described in EN13205, information was lacking in the reviewed articles.

#### 4.4.1. Test Conditions

In the reviewed articles, a wide variety of laboratory set-ups have been used by the authors. This includes different chamber dimension sizes and set-ups and different wind tunnel set-ups. This difference does not allow a direct comparison between articles to be undertaken. Twelve articles performed their experiments in a wind tunnel, six articles performed the experimental work in an aerosol chamber, five articles were performed in a test chamber or an air chamber, one article was performed in a long wall gallery [43] and one article used an outdoor environmental chamber [33].

Test condition requirements are set out in EN13205-2 [11] and EN13205-4 [12]. For inhalable fraction samplers, tests are required to be performed in an aerosol chamber or a wind tunnel. For those personal inhalable samplers for use in either environments with strong ventilation (>0.5 m/s wind speed) or outdoor environments, tests need to be performed either on a life-size mannequin or on a simulated torso (this needs to be stated by authors where this has been used). The reporting requirements for the experimental set-ups are for (a) the requirement for describing the test facilities including dimensions and a schematic diagram that also illustrates the sampler’s locations and (b) details on the velocity profiles, turbulence and blockage for wind tunnels.

In the reviewed articles, information about the dimensions of the chambers has not been discussed in two articles ([26,31]). Schematics of the chambers and/or wind tunnels with the location of the samplers is also generally not provided. An example of schematics that could be provided is illustrated in Aizenburg et al. [20] in which figures are provided for the experimental set-up and the positions of the samplers on the torso.

EN13205-4 [12] sets out the requirements for the set-up for laboratory sampler comparisons. As part of the reporting requirements, a description of the testing facilities with a schematic diagram to be provided. For example, a schematic of the set-up and sampler location is provided in [29].

#### 4.4.2. Particle Aerodynamic Diameter

The requirements of EN13205 (i.e., [11,12]), is that for the inhalable fraction needs to between 1 to 100 µm, the respirable fraction between 0.5 to 40 µm, and the thoracic fraction between 0.5 to 40 µm. There should also be up to three polydisperse aerosols which are selected to ensure the relevant range is covered. Polydispersion of the aerosols is discussed in Section 4.4.16; however, in many cases, the dispersion of the aerosols is not discussed in the articles.

For those articles that assessed the inhalable fraction (15 studies), fourteen of these studies had the particle aerodynamic diameter between 1 and 100 µm. However, for one article no information was provided on the particle aerodynamic diameter [29] for one of the aerosols used in the article. In this article, the particle aerodynamic diameter is provided for the ISO test dust, however, is not provided for the organic dust used.

For those articles that assessed the respirable fraction and that provide the particle aerodynamic diameter, these are between 0.5 and 40 µm, except for one article. In this article, the particle aerodynamic diameter was between 38 and 75 µm for coal dust [43]. Three articles do not provide a discussion for all the particle aerodynamic diameter for the respirable fraction used in the article [28,29,41].

For the three articles that assessed the thoracic fraction [31,32,33], the particle aerodynamic diameter are between 0.03 to 25 µm.

#### 4.4.3. Wind Speeds

Typically, wind speeds of 0.5 and 2 m/s have been used, with the range of wind speeds in the reviewed articles varying between 0.2 m/s to 4 m/s.

Wind speeds could have an impact on the flow rate and performance of the samplers. For example, the flow rates used by Aizenberg et al. [19] for the IOM, Button, GSP and 37 mm CFCs were dependent on the wind speed, with a higher flow rate used for the samplers (except for the Button sampler) at a wind speed of 2.0 m/s compared to a wind speed of 0.5 m/s. In addition, the wind speed affected the match to the inhalability convention used, with a better fit at the lower wind speed. Kenny et al. [22] also found that a low wind speed of 0.5 m/s, the IOM sampler agreed well with the inhalability convention used. Kenny et al. in another article [23] also showed that at higher wind speeds of 4 m/s, none of the inhalable samplers performed well at this wind speed.

The effect of the wind speed on sampler results has been specifically investigated in three of the articles [24,29,37] Reynolds et al. [24] concluded that for the compared samples, wind speed was one factor (along with the test aerosol) that affected the sampler comparison results. As an example, for the IOM/CFC Pearson correlation ratios for swine dust, this was 0.67 at a wind speed of 0 m/s, then decreased to −0.47 at 0.2 m/s and changed to 0.12 at a wind speed of 1 m/s. In contrast, O’Shaughnessy et al. [29] found that the bias ratio for the 37 mm CFC/IOM was not significantly different at 1 at two wind speeds (0.2 and 1.0 m/s). As illustrated by these results, the wind speed can impact the performance of samplers and could therefore impact in the sampler selection depending on the environment. Sleeth and Vincent [37] have shown that at lower wind speeds, the sampling efficiencies of the IOM, Button, GSP and the 37 mm CFC samplers improved.

In terms of the wind speed described in EN13205-2 [11], it is discussed in outdoor workplaces that a higher wind speed can be used than discussed in the standard (0.5 m/s to 4 m/s).

The wind speed for indoor workplaces in EN13025-4 [12] is that testing should be at a wind speed of 0.2 m/s or below. Only five articles test at this wind speed [25,29,33,35,37]. It would be beneficial for authors to clarify the reasoning for the wind speeds selected such as comparing against the conventions.

#### 4.4.4. Wind Direction

The requirements for EN13025-4 [12] for the wind direction is for the range to be an omnidirectional average with a continuous revolution or up to four stepwise values. For nearly half of the articles (11 articles; 44%), there is no discussion on the consideration of the wind direction. Generally, for those articles that provided information on the wind direction, information is lacking. For example, one article [31] only discusses that samplers were placed in a rotating table or that samplers were placed on a rotating mannequin [29]. One article does discuss omnidirectional sampling, in that using the mannequin and the set-up was similar to omnidirectional sampling [36].

#### 4.4.5. Aerosol Composition

The requirement of EN13205 is that the aerosol particles should be spherical (either solid or liquid) and be approximately isometric. Common aerosols used for assessing sampler performance has included aluminum oxide coal dust, Arizona Road Dust and glass microspheres amongst others.

In terms of the aerosol particles being spherical or approximately isometric, there is limited discussion in the published articles. Eighteen articles (72%) provide no information on this point. Glass microspheres are used in three articles [32,38,43]. For aluminium oxide where this is discussed, this is irregular shapes (i.e., [41]) or spherical for ammonium fluorescein (i.e., [41]).

This information is important for evaluating sampler performance/comparison and to understand the particles the samplers are challenged with, aerosol composition details (powder/liquid/shape) should be noted by authors.

#### 4.4.6. Aerosol Agglomeration

The majority of articles (22 articles; 88%) do not consider or discuss aerosol agglomeration. One article states that for aluminium oxide, there was an absence of agglomeration [20]; however, some aluminium oxide agglomeration was observed in another article [22]. For ammonium fluorescein, some agglomeration has also been observed [41]. Under EN13205-2 [11], it is assumed that the chemical composition of the aerosol would not be influential. However, agglomeration can be verified by a visual microscopic information of the test particles. It would be useful for authors to include information on agglomeration even if none is observed.

#### 4.4.7. Collected Mass and/or Internally Separated Mass

Under EN13205-2 [11], the amount of dust should not exceed the typical values in the workplace. This is an optional and overly broad requirement and is not discussed in any of the included articles.

#### 4.4.8. Aerosol Charge

Twelve articles provide no information on the aerosol charge. The aerosol charge was neutralised in eight articles. In three articles, it is stated that the aerosol charge was not neutralised. The requirement for aerosol charge in EN13205-2 [11] is that a neutralised aerosol should be used for non-conducting samplers and where possible to use samplers consisting of charged materials.

One article specifically investigates conductivity of the IOM sampler (conductive material) and the 37 mm CFC sampler (both conductive and non-conductive material). A higher sampler efficiency was observed for the conductive samplers, however no details on the aerosols were provided [34].

#### 4.4.9. Sampler Specimen Variability

Sampler specimen variability tests are only compulsory for EN13205-2 [11] for some respirable and thoracic fractions samplers only. These are defined in the following:

- For type A: EN13205-1:2014 [47], Clause 6 and EN13205-2 [11] and CEN/TR 13205-3 [48]. Additionally, one test aerosol according to EN13205-4:2014 [12] or EN13205-5:2014 [49], 5.2a and EN13205-6:2014 [50], Clause 5.
- For type B: EN13205-1:2014 [47], Clause 6 and EN13205-4 [12] and EN13205-6:2014 [50], Clause 5.
- For type C: EN13205-1:2014 [47], Clause 6 and EN13205-5 [47] and EN13205-6:2014 [50], Clause 5.

Within the standard [47], type A is a test to determine the performance for those size distributions at the largest range. Type B is a test that is based on three test aerosols, whilst type C is a test that is only used for the aerosol that would be present at the workplace for the test.

For this requirement, the test group should be as large as possible with up to six values. Fifteen articles (60%) consider the respirable and/or thoracic fractions. Three articles do not provide clear information. Chen et al. [27] and Görner et al. [25] provide no information on sampler specimen variability, whereas it is not clear in another article [40]. A variety of sampler specimen variability is employed in the reviewed articles, for example from six measurements for each sampler [32,38] to a total of 216 measurements performed in total [28].

#### 4.4.10. Excursion from the Nominal Flow Rate

This is only compulsory for EN13205-2 [11] for some respirable and thoracic fraction samplers. These are the same as those discussed in Section 4.4.9.

Where this is performed, the requirement is the flow rates should be within 5–10% range of the nominal flow rate and the nominal flow rate and flow rates (lower and higher) at one wind speed should be performed.

For articles that assessed the respirable and/or thoracic fraction, eight articles only used the nominal flow rates for the sampler. In a number of articles, the nominal flow rates are not used with no discussion from the authors on this, i.e., [36], and are also greater than 10% of the nominal flow rate [30].

#### 4.4.11. Flow Rates Applied

In Table 1 and the Supplementary Materials, the flow rates as stated by the authors are supplied. There are also cases in which the flow rates used are not stated by the authors (i.e., [22,34,42]). Where flow rates are modified, these should be stated by the authors and the manufacturers nominal flow rates at the time of the article should also be supplied.

Where authors have modified the flow rates from that recommended by the manufacturers, then the reason for doing so should be stated clearly. For example, Lee et al. [31] discuss that the flow rates used in their article were used to achieve a 50% sampler efficiency for a particle size of approximately 10 µm. Chen et al. [26] also discuss that the sampler flow rates have been modified to meet the requirements of the 50% cut size of the flow rates. However, for some of the articles where the flow rate has been modified, no explanation can be identified for modifying the flow rates (i.e., [22,28,39]).

Some information on recommended flow rates is publicly available from sampler manufacturers. For example, SKC supply flow rates for their manufactured samplers, such as the IOM and Button inhalable samplers. Casella supply certificate of conformities on their website which shows the sampler flow rate(s). However, technical reports for samplers can be limited and not publicly available. We would invite manufacturers to allow documentation for samplers to be publicly available and available to stakeholders.

#### 4.4.12. Wall Deposits

Verpaele and Butler [51] have discussed the current issues for “wall loses” which are defined as particles which are not collected on the filter but are deposited on other parts such as the sampler inlet. These are important to consider especially in the context of comparing with OELVs [52].

The contribution of wall deposits has been discussed in two articles [27,35]. For one of these articles, particle losses from wall deposits are considered in one article, but only for one of the samplers used [39]. The other articles discusses the loss of particles from wall deposits; however, this has not been considered as part of the analysis [35].

In the other reviewed articles, it has not been stated if wall deposits have been considered and accounted for. Given the importance of this issue it should be considered by authors in their experimental set-up, analysis and reporting moving forwards.

#### 4.4.13. Particle Collection Substrates Handling

The sampling substrate materials (also known in articles as the filter materials) used in the articles is described in the Supplementary Materials. Sampling substrates is defined in EN1540:2011 (Workplace exposure—Terminology) [53] as “*medium on which airborne chemical and/or biological agents are collected for subsequent analysis*” and it is also noted in the standard that for the collection of airborne particles; this includes filters, polyurethane foams and sampling cassettes.

In a number of cases, there is limited or no information supplied on the sampling substrate materials used for the analysis. In six of the reviewed articles [26,27,34,38,40,42]), no information is supplied on the substrate materials. Additionally, in a number of the reviewed articles (i.e., [23,25]) only partial information is supplied on the substrate materials. For example, information is not supplied for the sampling substrates used in all the samplers [23].

Specifically, information should also be provided on any handling procedures used for the sampling substrate materials as sixteen articles do not provide this information. Handling procedures could include placing filters in a climate controlled room [20], storing in a desiccator [37] or weighing the substrates in chambers with stable temperature, air and humidity [43].

It is important moving forward for both authors and reviewers that information is supplied for the sampling substrate materials (material used, diameter, pore size) used in all of the assessed samplers in the article. This is important is this can affect the evaluation of the samplers.

#### 4.4.14. Weighing

EN13205 provides a reference to ISO 15767 [54] for weighing aerosol mass. Within ISO 15757, weighing requirements discussed include using blanks for correcting for weight instability, the balance to be used (a resolution of either 1 µg or 10 µg is recommended), and the environmental controls to be used for weighing.

Of the 25 articles, 14 articles do not supply information on balance used, although this in some cases may not be relevant due to the analysis technique used (e.g., [28,38]). However, if for example the comparison is based on a specific metal, then other requirements need to be fulfilled, such as those in EN 13890 [55]. However, for those articles that have described weighing (balances used), the information can be lacking. In some cases, only the balance name is provided [32,34,36]. For example, in four articles information is supplied that an ultra-microbalance is used with the resolution of the balance provided.

Moving forward, it would be beneficial for information to be provided by authors and requested by reviewers on the balance used, resolution for the balance (such as a resolution of 1 μg or 10 μg has been used) as these are important parameters for analysing the results.

#### 4.4.15. Temperature

Under EN13205-2 [11], the temperature for the experiments should be in the range of 15–25 °C and that the temperature during the tests should be detailed. However, information on the temperature is only provided in three articles (12%). Lee et al. [31] performed their experiments at a temperature of 21–23 °C and Sleeth and Vincent discuss that the mannequin used for sampling was heated to 33 °C [37]. Teikari et al. [33] who used an outside environmental chamber discuss this was performed in summer conditions, where the temperature is between 12 and 22 °C.

Details on the temperature used for the sampler experiments should be recorded ad provided by authors or requested by reviewers moving forward.

#### 4.4.16. Pressure

Only one article has provided information on the pressure. This article only discusses that the pressure inside of the test chamber used was equal to that of the external air pressure [23].

Moving forward, details of the pressure used during the sampling testing should be provided as detailed in EN13205-2 [11].

#### 4.4.17. Aerosols Used

Under EN13205 there are requirements discussed for the test aerosol to be used in EN13205-2 [11] and EN13025-4 [12] as follows:

- EN13205-2 (Laboratory performance test, based on determination of sampling efficiency): For assessing respirable and thoracic samplers, the GSD for monodisperse aerosols is required to be less than 1.1. For assessing inhalable samplers, the GSD for near-monodisperse aerosols is required to be less than 1.3. In terms of aerosol composition, the aerosol (either monodisperse and/or polydisperse) can be spherical or approximately isometric.
- EN13205-4 (Laboratory performance test based on comparison of concentrations). This includes the requirement for selecting test dusts which can produce three polydisperse test dusts) and also that particles are required to be spherical (either solid or liquid) or approximately isometric. There is also the requirement for the particle aerodynamic diameter with the ranges being 1 to 100 µm for the inhalable fraction, 0.5 to 40 µm for the thoracic fraction and 0.5 to 15 µm for the respirable fraction.

Fourteen of the reviewed articles (56%) provide no information on the dispersion (monodispersed or polydisperse) of the aerosol. Two articles [19,23] used near-monodispersed articles, and three articles used monodisperse aerosols [27,35,41] with the remaining six articles using polydisperse aerosols.

Information is generally lacking for the GM and GSD for the aerosols used. The GM of the test aerosols is not reported in four of the reviewed articles where the aerosol dispersion is discussed. The GSD of the test aerosol used is not reported in nine of these articles. There are also four of the articles which supply information on the GM of the test aerosol but do not provide information on the GSD. In one article, the composition of the test aerosol is not discussed [34].

There is a need, going forward, to consider if studies meet the requirements for sampling performance for aerosol requirements and for publications to explicitly address the following questions:

- Is the GM and GSD of the test aerosol provided?
- Does the GSD of the test aerosol meet the requirements for the sampler fraction in the standard?
- Is the dispersion of the test aerosol stated (monodisperse, nearly monodisperse, polydisperse) and does this meet the requirements of the standard for the fraction(s) being assessed and the nature of the test?

#### 4.4.18. Correction Factors for Particle Shape

Only two articles considered correction factors for particle shape. However, it is discussed within EN13205, i.e., in EN13205-2 [11] that correction factors for particle shape for each aerosol should be calculated for each aerosol where this is used. Kenny et al. [23] and Gudmundsson and Lidén [40] discuss that correction factors were not required. In terms of EN13205-2, the requirements are to document the correction factors where used.

#### 4.4.19. Correction Factors for Particle Density

Eighteen articles do not consider correction factors for particle density. One article provides the density, with no other details provided [39]. One article also discussed that this was not required [40]. Lee et al. [41] and Maynard and Kenny [38] performed a correction for the density for the APS, with Maynard and Kenny also supplying the density of the aerosol (within 0.5% of the manufacturers supplied density of 2.45 g/cm^−3^.

In terms of EN13205-2, the requirements are to document the correction factors where used.

#### 4.4.20. Determination of Test Aerosol Concentration

Nine articles (36%) provide no discussion on determining the concentration of the test aerosol. Within EN13205-2 [11], there is a requirement to use isokinetic sampling for determining the test aerosol concentration. Six articles use isokinetic sampling for determining the aerosol concentration. Specifically, probes are used [19,36] and samplers used [22,35,37] in these articles. Other methods used include sedimentation collection plates [20] and by ELPI [33].

Test aerosol concentrations are an important factor that is needed to be known for comparing between different samplers. It is important that this information is provided by authors or requested by reviewers, as this is not reported in a number of articles as discussed.

#### 4.4.21. Position and Orientation of the Sampler

In EN13205-2 [11], it is required that the position and orientation is noted. It was encouraging to note that 24 articles (96%) provided some discussion on the position and orientation of the samplers under review.

#### 4.4.22. Pumps Used and Their Volumetric Flow Deviation

Information on the pumps used for sampling and also their volumetric flow deviation is limited; for example, 15 articles provide no details on the pumps used or their volumetric flow deviation. Seven articles provide information on pumps but not on the volumetric flow deviation. Only three articles provide some discussion on the pumps used and their volumetric flow deviation [20,28,33].

Moving forward, it would be beneficial for authors to include this information in articles as outlined in EN13205-2 [11], such as ensuring the flow rate is with 2% of the intended flow rate and the method used to verify this.

### 4.5. Limitations Identified by Authors of the Reviewed Articles

Five articles have identified limitations in their obtained results. Aizenberg et al. [21] identified that further discussion of performance (accuracy, bias and precision) than that beyond which is discussed in their article was not possible due to the limited number of aerosol particle sizes and wind velocities used in the article. Maynard [32] identified that using polydisperse aerosols for characterising the sampler penetration for diameters of 20 µm was in its infancy at the time of the article (published in 1999). This would result in issues for aerosol stability, the aspiration efficiency and the detection of sufficient particle numbers. This in turn could lead to large errors for large particle diameters.

Maynard and Kenny [38] discuss a limitation of their article that investigated Casella samplers, in that there was an issue with a tolerance error of 13% on the cyclone input slot width. This has since been rectified by the manufacturer. Patts et al. [43] identified that their article was only limited to a relative sampler comparison article as for measuring aspiration efficiency for each inlet (dust diameter and air velocity function); then, a well-controlled wind tunnel which is equipped with real time aerosols should be required. Reynolds et al. [24] identified limitations with the concordance between the MMADs produced in the laboratory and the field. This limited their article to investigating wind speed effects on dust types in the laboratory.

## 5. Conclusions

A narrative review has been performed for laboratory sampler efficiency, performance and comparison studies published between 1994 and 2021, with 25 articles identified as being relevant.

From this review, it has not been possible to align the articles and to provide an overall image of the articles and to provide conclusions on sampler performance due to the issues discussed in the articles and summarised below. It is clear that there is a lack of standardisation for performing these testing experiments. The benefit of this review, however, is that it is aimed to start discussions amongst the community and to allow standardised methods to be advanced which take into account the factors discussed in this section going forward. This would then allow the potential performance/efficiency of sampler(s) to be compared across multiple studies.

A number of aspects have been highlighted in this article to be taken into account moving forward, with many of these also being highlighted in our previous review (Hanlon et al., 2021 [13]). Samplers tested need to be clear (such as using the names as reported by the manufacturers), flow rates used to be reported, along with more detailed information on chambers and wind tunnels used. There is a need for nominal sampler flow rates to be stated and where flow rates have been modified for the reasoning to be clearly detailed. For the aerosols used, the GSD and GMD of the particle sizes, the aerosol dispersion and the aerosol used need to be clearly stated, with a need that the aerosols used comply with the standards such as EN13205. Where the standard is not met for the test aerosols; then, it should be considered if the article can be considered for further analysis. There is also the need to report on substrate conditioning, balances used (and specifications) and correction factors (where used) amongst others. The full reference if the conventions also need to be stated by authors.

Moving forward, it would be beneficial for follow-up studies to be carried out, i.e., evaluating the sampler performance over a period of years so that the performance of sampler(s) can be re-evaluated. For this to occur, accurate recording of the samplers used is required such as year purchased, batch number (where available) and nominal flow rates of the samplers. If unexpected results are obtained, these should be further investigated such as was the case, for example, in the article by Maynard and Kenny [38]. This illustrates the need for all stakeholders to be involved. Standardisation organisations (such as ISO) should also be involved in discussions to ensure there are no gaps, etc. in the standards.

In terms of reviewing articles, reviewers should ensure that all required information is supplied by authors, such as nominal flow rates, the reasoning behind modifying the nominal flow rates. GM and GSD of the test aerosols and detailed information on the set-up is provided.

There is a need for more rigorous, documented and systematic testing for laboratory testing of samplers. This also involves the need for all stakeholders to work together to advance the field.

## Figures and Tables

**Table 2:** Aerosols and conventions used in the included articles.

Aerosol Used	Fraction	GM (GSD) of Aerosol (µm)	Samplers	Conventions Used	Publication Date	Reference
Agriculture *	Inhalable	Swine: 10.3–17.9 (5.6–8.6)	IOM	None stated	2009	[24]
		Chicken: 7.6–33.0 (2.6–4.2)	Button			
		Turkey: 3.9–13.6 (3.1–4.3)	37 mm CFC			
Aluminium oxide	Inhalable	7, 29, 70 µm (1.35)	IOM	ACGIH (1999)/CEN (1993)/ISO (1995)	2000	[19]
			Button			
			GSP			
			37 mm CFC			
	Inhalable	MMAD: 70 (1.35)	IOM	ACGIH (2000)/CEN (1993)/ISO (1995)	2001	[20]
			Button			
			GSP			
		MMAD: 7, 29 and 70 (GSD not supplied)	IOM	ACGIH (1999)/CEN (1993)/ISO (1995)	2000	[21]
			Button			
			GSP			
			37 mm CFC			
		6–90 (1.2–1.4)	IOM	ACGIH (1985)/CEN (1993)/ISO (1995)	1999	[22]
			Seven-hole			
			GSP			
			37 mm CFC			
		1–25 (1.53–5)	IOM	CEN pre-standard (1995)	1997	[23]
			Seven-hole			
			GSP			
			PAS-6			
			PERSPEC			
			CIP-10l			
			37 mm OFC			
			37 mm CFC			
	Inhalable	MMAD (wind speeds of 0.10 m/s, 0.24 m/s and 0.42 m/s); F1200 (9.6, 9.5, 9.3) F800 (13.9, 12.8, 12.4) F500 (28.8, 32.7, 28.7)	IOM	ISO (1992)/CEN (1992)/ACGIH (1985)	2012	[37]
			Button			
			GSP			
			37 mm CFC			
	Respirable	MMAD of 4.45 and 2.86 µm	CIP10R	ISO/CEN (2002)/ACGIH (2006)	2010	[41]
			GK2.69			
			FSP10			
			25 mm open cowl (ref)			
Ammonium fluorescein	Respirable	2.1–14.6 µm (0.1–0.5)	GK2.69	ACGIH(2014)/CEN(1993)/ISO (1995)	2016	[31]
			GK4.126			
			FSP10			
	Respirable	Not discussed	CIP10R	ISO/CEN/ACGIH	2010	[41]
			GK2.69			
			FSP10			
			25 mm open cowl (ref)			
Arizona Road Dust	Respirable	2.8 and 4.6 (GSD not presented)	GK2.69	None stated	2014	[30]
			FSP 10			
			CIP10R			
			SIMPEDS (ref)			
	Respirable	1 m/s: 17.4 (1.86) 2 m/s: 22 (1.98)	GK2.69	ISO/CEN/ACGIH discussed	2016	[39]
			FSP10			
			CIP10R			
			PPI8			
			SIMPEDS (ref)			
Coal Dust	Respirable	Close to 15 µm (1.8)	10 mm Dorr–Oliver cyclone	CEN (1993)/ISO (1995)/ACGIH (1994–95) respirable convention	2001	[25]
			SKC Plastic cyclone			
			Casella Plastic cyclone			
			SKC Aluminium cyclone			
			South Africa Cyclone			
			Lippmann 6 mm cyclone			
			ODPN Cyclone			
			CXF-2 virtual impactor			
			MRE 113A			
			CIP 10-R			
			C.A.Th.A. sampler			
			Lippmann 12 mm cyclone			
	Inhalable	7.8 (GSD not stated)	GK2.69	ISO/CEN/ACGIH discussed	2016	[39]
			FSP10			
			CIP10R			
			GK4.16			
			PPI8			
			SIMPEDS (ref)			
	Respirable	Not stated	10 mm Dorr–Oliver	None stated	2014	[28]
			SKC Aluminium cyclone			
			BGI4L cyclone			
			GK2.69			
	Inhalable and respirable	53 and 38–75 (no GSD stated)	IOM inhalable	None stated	2017	[43]
			IOM-MOD			
			Zefon coal dust cassette			
Glass microspheres	Respirable	MMAD of 24 µm and GSD of 1.4 µm in horizontal wind tunnel and MMAD of 27.5 µm (GSD of 1.6) in vertical calm air tunnel	IOM	CEN (various years)-ISO (1995)-ACGIH (1994–1995)	2010	[36]
			CIP10-l			
			37 mm CFC			
			37 mm CFC with ACCU-CAP insert			
			Button			
	Respirable	4.0 (2.2)	Metal bodied cyclone (Casella)	ISO/CEN/ACGIH discussed	1995	[38]
			Conductive plastic cyclone (Casella)			
			BGI cyclone			
	Thoracic	1–25 (1.75–4)	CATHIA	CEN (1993)/ISO (1995)	1999	[32]
			Modified cowled			
			IOM thoracic			
			GK2.69 thoracic			
			Modified SIMPEDS			
			Modified IOM inhalable			
Glass (Ballotini beads)	Thoracic	MMAD < 20 µm	GK2.69	ACGIH(2014)/CEN(1993)/ISO (1995)	2016	[31]
			GK4.126			
			FSP10			
ISO test dust	Inhalable and respirable	MMAD: ISO-still air: 1.37–7.28 (2.17–4.14) ISO-0.2 m/s: 2.23–9.17 (2.02–3.90) ISO-1.0 m/s: 2.01–10.10 (2.01–9.77)	IOM	Not stated	2007	[29]
			37 mm CFC			
			SKC Aluminium cyclone			
Organic dust **	Inhalable and respirable	Not stated	IOM	Not stated	2007	[29]
			37 mm CFC			
			SKC Aluminium cyclone			
Polydisperse polystyrene	Respirable	Count mode: 0.7 (1.5)–4.6 (2.3)	Graphite filled Casella	Not stated	1998	[40]
			BGI stainless steel			
			BGI carbon steel			
			SKC Aluminium cyclone			
Potassium sodium tartrate	Respirable	30–40	Nylon cyclone	ISO (1993)/CEN (1992)/ACGIH (1998)	1999	[26]
			SKC Aluminium cyclone			
			New foam sampler			
	Respirable	4 (2.2)	10 mm Dorr–Oliver cyclone	ACGIH/ISO/CEN (years unclear)	1994	[42]
			Higgins–Dewell cyclone			
	Respirable	3.5 (1.3) and 7.4 (1.5)	10 mm nylon cyclone	ACGIH/ISO/CEN respirable convention	1999	[27]
			SKC Aluminium cyclone			
			Multi-Inlet cyclone			
			Big Body cyclone			
Quartz	Inhalable, respirable and thoracic	0.03–10 (no GSD provided)	Modified IOM IOM (ref)	Not stated	2003	[33]
			37 mm CFC			
			37 mm OFC			
			Respicon			
			Two stage cascade impactor			
Refractory mineral dust (Mullite and 10% crystalline silica)	Respirable	17.4 (no GSD reported) mass median	GK2.69	None stated	2014	[39]
			FSP10			
			GK4.162			
			PPI			
Sodium fluorescein	Inhalable	6 (no GSD reported)	IOM	Draft CEN (1995)	1997	[23]
			Seven hole			
			GSP			
			PAS-6			
			CIP10-l			
			37 mm OFC			
			37 mm CFC			
	Inhalable	17, 26, 38 (no GSD provided)	25 mm CFC	None stated	1995	[35]
			New inhalable sampler			
Test aerosol (not stated)	Inhalable	MMAD 7 (no GSD stated)	37 mm CFC	None stated	2003	[34]
			IOM sampler			

Key: GM = Geometric Mean. GSD = Geometric Standard Deviation. MMAD: Mass Median Aerodynamic Diameter. For samplers with (ref), these were used as reference samplers. Information on isokinetic sampler is provided in the Supplementary Materials. * For this article, the authors used agricultural dust in a still air chamber and a wind tunnel for providing information on the wind speed effect on sampler performance along with field experiments. ** For this article, the authors used organic dust and ISO test data for demonstrating the differences between types of dust.

**Table 3:** Respirable dust concentrations ratio when compared with a SIMPEDS sampler.

Sampler	Manikin Facing the Emission Source	Manikin Side on to the Emission Source	Manikin Rotating
GK 2.69	1.00	0.95	0.93
PPI8	1.06	1.20	1.19
GK 4.162	1.09	1.03	1.07
FSP10	1.08	1.08	1.09

## Data Availability

Data supporting the reported results is available as Supplementary Material.

## Supplementary Materials

The following supporting information can be downloaded at https://www.mdpi.com/article/10.3390/ijerph20010267/s1, Table S1: Data extraction for Laboratory-Based Aerosol Sampler Efficiency, Performance and Comparison articles.
